# A simple and rapid method for fish sex identification based on recombinase-aided amplification and its use in *Cynoglossus semilaevis*

**DOI:** 10.1038/s41598-021-89571-z

**Published:** 2021-05-17

**Authors:** Zhichao Nie, Peng Lü, Rusong Zhang, Yishuai Tu, Zhenni Liu, Yin Li, Cong Tang, Xiqing Li, Kun Zhao, Qiang Zhou, Feng Li, Jun Wang, Zhanzhuang Zeng, Min Tu, Hong Zhang

**Affiliations:** 1 Anhui Provincial Key Laboratory of the Conservation and Exploitation of Biological Resources, College of Life Sciences, Anhui Normal University, Wuhu, 241000 China; 2 Institute of Life Sciences, Jiangsu University, Zhenjiang, 212013 China; 3 Anhui Microanaly Gene Co., Ltd., Hefei, 230601 China; 4 Waksman Institute of Microbiology, Rutgers, The State University of New Jersey, Piscataway, NJ 08854 USA; 5 Freshwater Fisheries Research Institute of Fujian Province, Fuzhou, 350002 China; 6 College of Life Sciences, Fujian Normal University, Fuzhou, 350002 China; 7 Present Address: The Genetic Engineering International Cooperation Base of Chinese Ministry of Science and Technology, The Key Laboratory of Molecular Biophysics of Chinese Ministry of Education, College of Life Science and Technology, Huazhong University of Science & Technology, Wuhan, 430074 China

**Keywords:** Biological techniques, Zoology

## Abstract

Fish sex identification is a basic technique of great importance for both fish genetic studies and fisheries. Due to the sexual reversal phenomenon in many fish species, a simple and rapid molecular identification method for fish genetic sex is urgently needed to suit versatile detection scenarios, such as point-of-need applications. In this study, we took *Cynoglossus semilaevis* as an example, established a recombinase-aided amplification (RAA)-based method for sex identification, and combined the RAA-detection with two result visualization approaches with distinct features, capillary electrophoresis (CE) and lateral flow dipstick (LFD). Specific primers and probe were designed to specifically detect the sex chromosome W of *C. semilaevis* in order to distinguish the genetic sex between males, pseudo-males and females. To evaluate the performance of our methods, the genetic sex for twenty-eight males, sixty-eight pseudo-males and fifty-four females were examined with the RAA-based method and classical PCR-based genotyping method, demonstrating the consistent results of sex identification between both methods. The RAA-LFD method is operationally simple, rapid (~ 30 min) and holds great potential for point-of-need applications of fish sex identification, including fishery fields. The method presented here could be effective for identifying fish gender with the ZW karyotype.

## Introduction

Males and females can differ strikingly in morphology, and reproductive development behavior. While these phenomena, known as sexual dimorphism, are widespread in many species, the genetic mechanisms regulating such a ubiquitous pattern are diverse. Among animals, fish becomes a paradigmatic model, because their sex-determination mechanisms vary from environmental factors to distinct modes of genetic controls^1^. Therefore, understanding of the genes and mechanisms involved in fish sex determination and reversal have long been one of the central topics in evolution and developmental biology. Molecular identification of fish genders is not only a necessary step in genetic studies related to sex determination and/or differentiation^2^, but also has broad applications in fishery industry.

Chinese half-smooth tongue sole (*Cynoglossus semilaevis*) has been recognized as an ideal model for fish sex determination and identification because of its typical mode of sex determination, the small genome and relatively large amount of studies on sex-determination genetics^1, 3^. Another key factor for *C. semilaevis* as a research model of sex identification is the sex reversal phenomena. *C. semilaevis* males have the ZZ sex chromosomes, while females have the ZW sex chromosomes and grow faster and bigger than males^4, 5^. Some *C. semilaevis* individuals carrying the ZW chromosomes become the pseudo-males, which show male physiology and morphology and produce only pseudo-male offspring if they are used as the male parent^1, 6, 7^. Owing to the high proportion of pseudo-males in the *C. semilaevis* population and the necessity for sex control, it is of great significance to distinguish pseudo-males from males and females. To address this, the *C. semilaevis* genome laid a solid basis for understanding sex-determination mechanisms and developing sex identification methods^1, 8^. On one hand, numerous genes related to sex determination and/or differentiation have been found, including DMRT1^1, 9, 10^, Dazl^11–13^, piwil2 ^14^, Figla^15^, GATA4^16^, aqp1aa^17^, and β-catenin1^18^. On the other hand, extensive efforts have been made to develop specific markers for *C. semilaevis* sex identification. Seven sex-specific amplified fragment length polymorphism (AFLP) markers was reported with one being further developed into a polymerase chain reaction (PCR) marker^19^. The microsatellite marker CseF-382 and a co-dominant marker CyseSLM were reported for sex identification^20, 21^. Through the construction of a high-density microsatellite genetic map of *C. semilaevis*, a number of sex-linked simple sequence repeat (SSR) markers were identified, and five of the markers were confirmed to be associated with sex^2^. More recently, new technologies, such as single nucleotide polymorphism (SNP), Insertion/deletion polymorphism (IDP) and penta-primer amplification refractory mutation system (PARMS) have been developed as fish sex identification methods^21, 22^.

**Figure 1:**
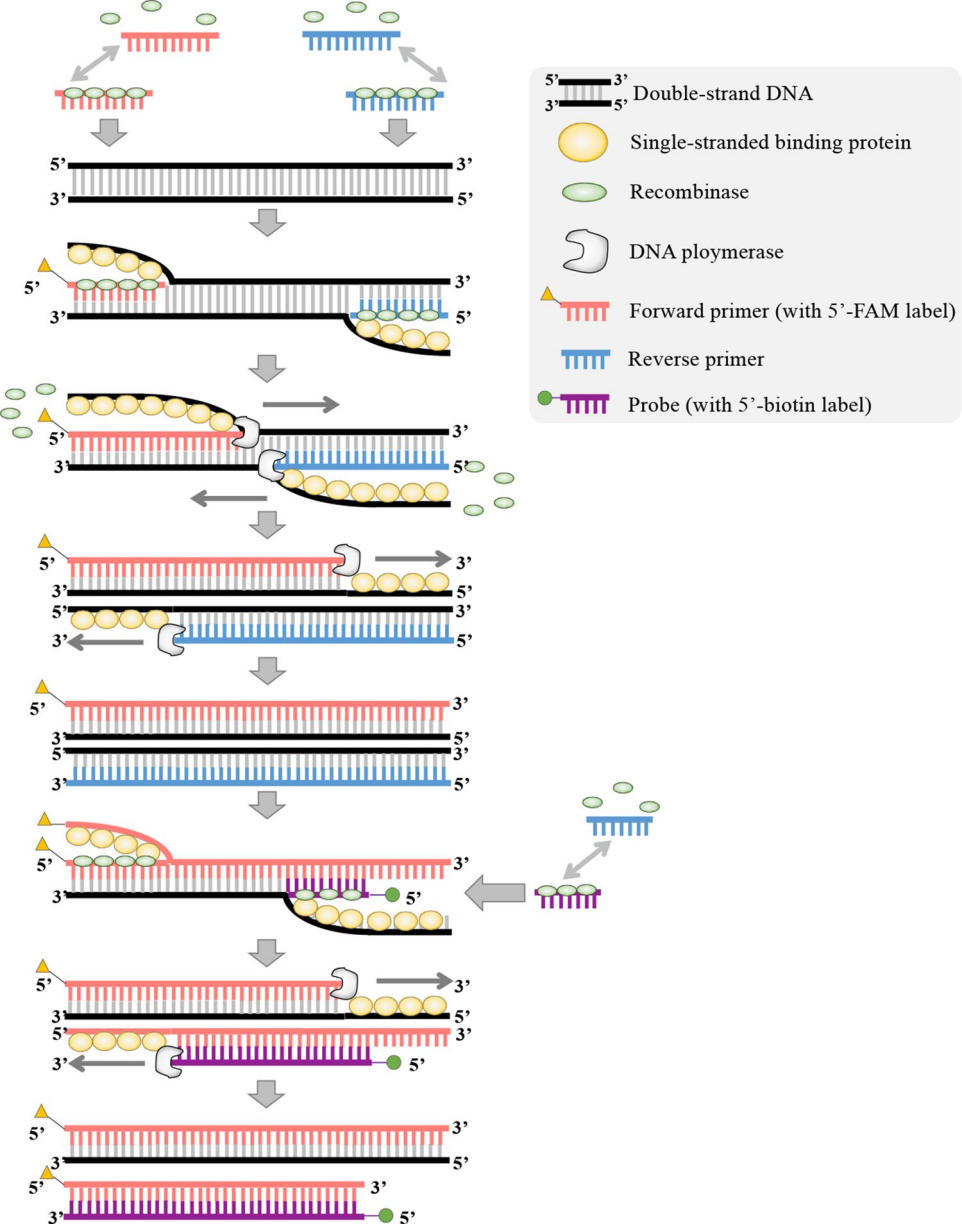
Diagram for the reaction mechanism of RAA. (1) The recombinase forms the nucleoprotein complex with a primer/ probe; (2) the primer- recombinase complex recognizes the homologous sequence in the template (double-stranded DNA) and hybridizes with the homologous site; After complex invasion, the SSB protein stabilizes the single complementary strand, while the primer hybridizes with the template strand; (3) the recombinase disassembles with the primer, and the DNA polymerase starts to synthesize DNA at the 3’ end of the primer; (4) DNA synthesis occurs from both strands leading by the forward and reverse primer, respectively; (5) Two double-stranded DNA are synthesized with the one initiated by the forward primer is labeled by FAM; (5) the probe can be hybridized with the double-stranded DNA template aided by the recombinase nucleoprotein complex; (6) Due to the probe’s specificity to the W chromosome, the amplification using the primer F and probe as forward and reverse primers, respectively, occurs only when the C. semilaevis fish contains the W chromosome; (7) DNA synthesis starts from both directions simultaneously when both the forward primer and the probe incorporated with the amplified double-stranded DNA, producing a shorter DNA product (147 bp) labeled by FAM and biotin at its 5’ end and 3’ end, respectively.

One limitation of these sex identification methods is that they are based on PCR and need gel electrophoresis for result visualization, which requires molecular biology lab conditions and expensive equipment, and are laborious and time-consuming. Recent advances in isothermal nucleic acid amplification technologies have revolutionized the conditions and requirements for nucleic acid detection. These methods, such as rolling circle amplification (RCA)^23^, strand displacement amplification (SDA)^24^, nucleic acid sequence-based amplification (NASBA)^25^ and loop mediated isothermal amplification (LAMP)^26^, eliminate the repeated heating and cooling steps of PCR, simplify incubation temperature and reduce amplification times. Due to the simplification of amplification conditions, these isothermal methods provide opportunities to performing amplification outside of laboratories, in low-resource settings and to developing a variety of applications in clinical medicine, agriculture, fishery and ecology^27–29^. LAMP-based rapid sex identification methods have been reported in fish and birds^30, 31^. Recombinase polymerase amplification (RPA)^32^ is another isothermal amplification method that have been widely used and adapted to hundreds of reported applications in recent years^33^. Compared to other isothermal amplification methods, RPA has several advantages: lowered amplification temperature (37–42 °C), shorter assay time (20–40 min compared to more than 60 min for other methods), elimination of the denature step, a small number of primers required and relatively simple primer design criteria^29^. Another method recombinase-aided amplification (RAA)^34^ shares essentially the same concept and methodology with RPA but uses different enzymes and temperature (its reaction mechanism shown in Fig. 1, detailed method described in “Materials and methods”). To establish a rapid and simple method for fish sex identification, we combined RAA with two result visualization approaches, capillary electrophoresis (CE) and lateral flow dipstick (LFD) using *C. semilaevis* as an example. Since the emphasis of this study was not to find novel sex-linked markers, a genomic region on the *C. semilaevis* sex chromosomes, where several sex identification markers have been identified and confirmed previously^2, 22^, was chosen as the target region to develop the RAA-based method. We demonstrate the new sex identification method is effective, validated by using previously established PCR-based genotyping.

## Results

### Establishment of the sex identification methods

**Figure 2:**
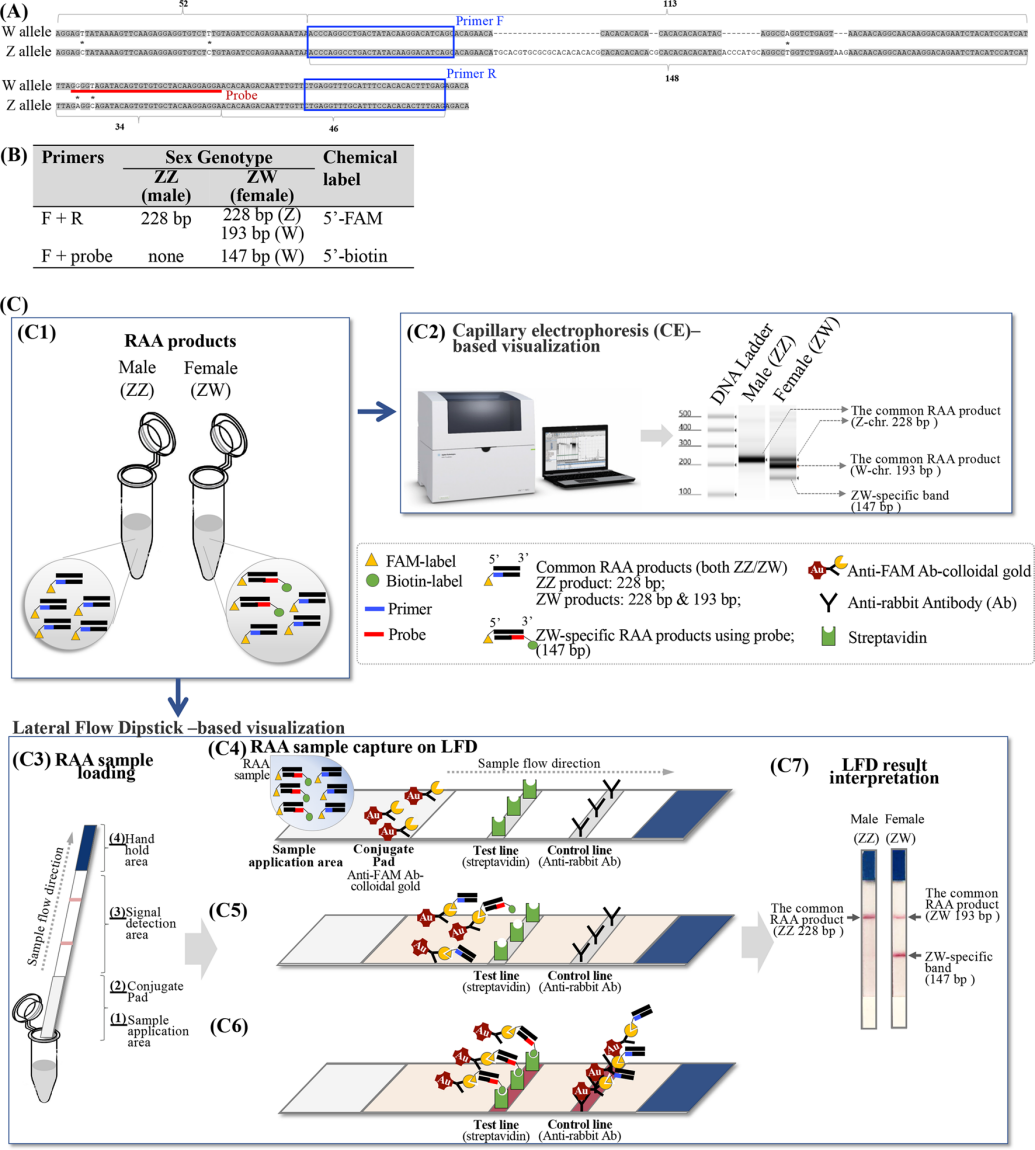
Development of the fish sex identification method using recombinase-aided amplification based lateral flow dipstick (RAA-LFD) assay. (**A**) Sequence alignment of the ~ 300-bp genomic region on both Z and W sex chromosomes used for RAA amplification, which is located within an ~ 1500-bp genomic region previously reported for sex identification^2, 22^. This region contains a few insertion and deletion polymorphisms between the Z and W chromosomes. Primer pairs F and R (highlighted in blue boxes) amplify 228-bp and 193-bp products for the Z and W sex chromosomes, respectively, while the probe and primer F specifically amplifies a 147-bp product for the W chromosome (the probe highlighted in red line). The SNPs are indicated using asterisks. (**B**) The primers and probe used for distinguishing sex marker between the Z and W chromosomes and their amplification products. (**C**) Schematic diagram shows that the amplification products of 228-bp (from Z chromosome), 193-bp (from Z chromosome) and 147-bp (from W chromosome) could be detected through CE (**C2**) and LFD (**C3** to **C7**). The LFD test strip contains four areas: #1. The sample application area where RAA reaction mixture is loaded, #2. the conjugate pad where RAA amplicons in the mixture are moving forward driven by capillary force and conjugated with immuno gold, #3. The signal detection area where the immuno gold-conjugated amplicons interact with the test line and the control line, #4. Hand hold area. Particularly, the 228-bp and 193-bp RAA products are captured on the control line of LFD test strip, while the 147-bp W-specific RAA product is captured through streptavidin–biotin interaction on the LFD test line.

An approximately 1500-bp intergenic region of the *C. semilaevis* sex chromosomes Z and W was chosen for RAA amplification and for establishing the RAA-based fish sex identification method, because two sex identification markers have been reported previously in this region^2, 22^ (Fig. 2A). A pair of RAA primers (F + R) were designed to amplify a 228-bp and 193-bp fragment from the Z and W chromosome, respectively, while an internal probe was designed to obtain a 147-bp short amplicon specific from the W chromosome (Table 1, Fig. 2A,B). Because the forward primer and the probe are labeled by FAM and biotin at their 5’ends, respectively, the amplicons can be captured by LFD and separately detected on the control and test lines (Fig. 2C). Two methods, RAA-capillary electrophoresis (RAA-CE) and RAA-lateral flow dipstick (RAA-LFD) were used to compare RAA result visualization in this study.

**Table 1:** Primers and probe sequences of RAA-LFD for *Cynoglossus semilaevis* sex determination.

Primer and probe name	Sequences
Primer F	5′-6FAM-ACCCAGGCCTGACTATACAAGGACATCAGC-3’
Primer R	5′-CTCTCAAAGTGTGTGGAAATGCAAACCTCAG-3′
Probe	5′ Biotin-TCCTTGTAGCACACACTGTATCTACCCC-3′

F: Forward primer, R: Reverse primer. 5′ ends of the probes were labeled with biotin, 5′ ends of the forward primer was labeled with FAM fluorophore.

### Performance of the RAA-based methods on fish sex identification

**Figure 3:**
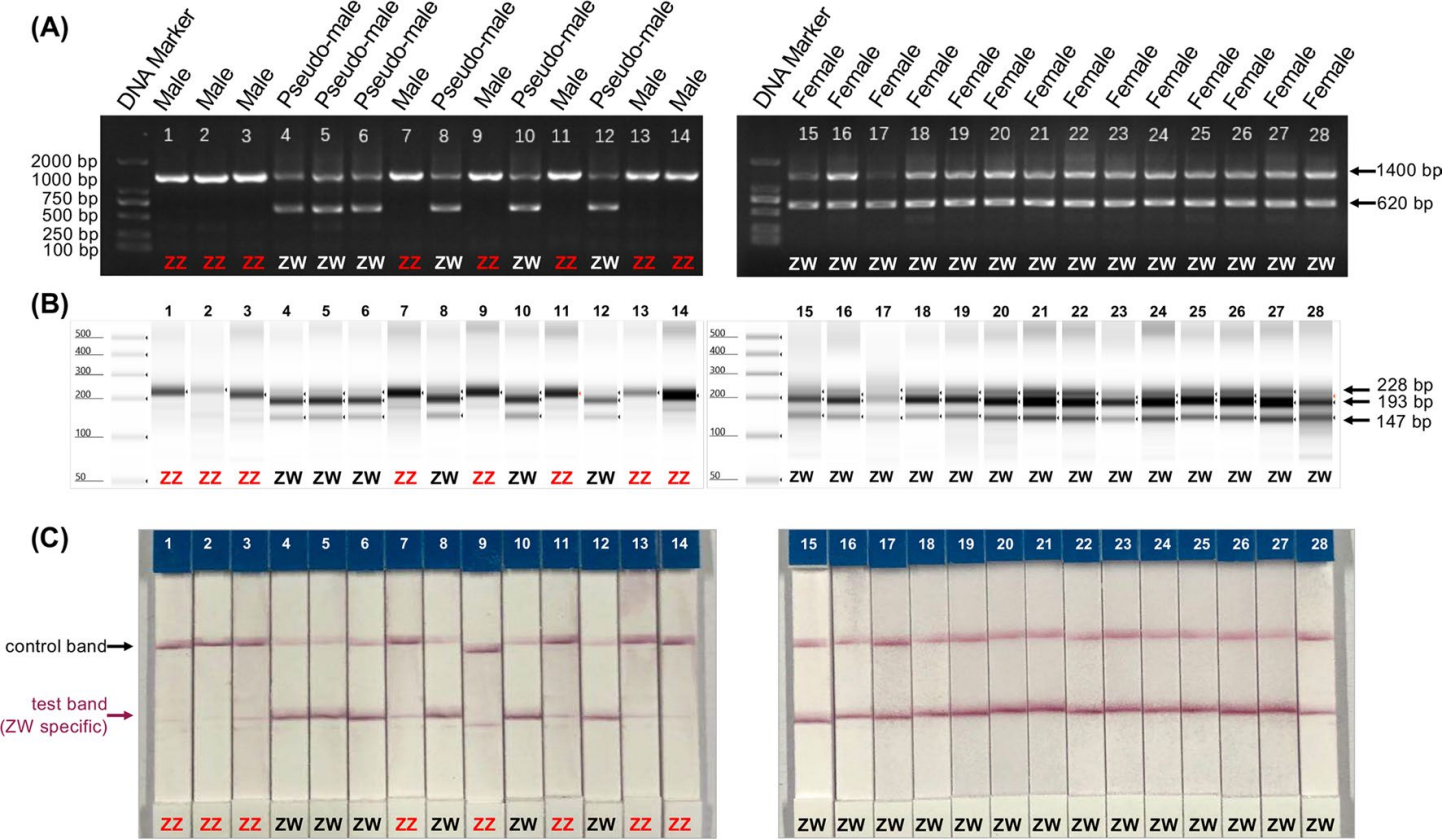
The RAA amplification coupled with either CE- or LFD-based visualization shows consistent sex identification results with those determined by IDP marker or based on fish gonads. (**A**) Genomics PCR results using the CS-SEX-3 primers for the twenty-eight fish specimens, with the genders determined by their gonads and labeled in the figure. The twenty-eight fish specimens include six pseudo-males (ZW karyotype, lanes #4, #5, #6, #8, #10 and #12). (**B**) CE results showed a single band of 228-bp for the male fishes (ZZ karyotype) and three bands for the female and pseudo-male fishes, including a 193-bp band and a 147-bp band specific for the ZW karyotype. (**C**) LFD visualization effectively captured and separated the W-specific, 147-bp RAA products from those bands amplified by the primer F and R (228-bp and 193-bp), facilitating the rapid and simple sex identification of *C. semilaevis*. All of the samples with ZZ chromosomes are highlighted in red.

A total of 150 fishes, consisted of 28 males, 54 females and 68 pseudomales, were used to test the effectiveness of the RAA-CE and RAA-LFD methods. These fishes came from two populations with their detailed information described in the “Animal samples” section in “Materials and methods”. The sex of fish was examined based on the type of gonad. The genetic sex identification results for population 1 and 2 are given in Fig. 3 and Supplementary Fig. 1, respectively. As shown in Fig. 3A, the PCR-based method using the IDP marker (primer pair CS-SEX-3F and 3R)^22^ successfully distinguished ZZ and ZW sex chromosomes. A single 1.4-kb PCR band was detected for all of the male fishes (ZZ karyotype) and an additional 620-bp band was amplified in the pseudo-male and female samples (ZW karyotype) (Fig. 3A and Supplementary Fig. 1A,D,G). The sizes of the IDP marker-derived PCR products are consistent with the previous report^22^. In parallel, a single 228-bp band was detected and visualized by using RAA-CE from all the male samples, while three bands, including a 228-bp, a 193-bp and a 147-bp, were observed for the pseudo-male and female samples (Fig. 3B and Supplementary Fig. 1B,E,H). The 193-bp and 147-bp bands were amplified from the W chromosome, while the 228-bp band was amplified from the Z chromosome (Figs. 2B and 3B). Thus, the RAA results are consistent with those of the classical PCR-based method. For the RAA-LFD method, all of the male fishes produced a strong control band on the LFD tests, while both control and test lines were observed for the pseudo-male and female samples (Fig. 3C and Supplementary Fig. 1C,F,I). The RAA-LFD results met its experimental design: male fishes with homozygous Z chromosomes produce the FAM-labeled 228-bp band, which will be captured by the control line; female and pseudo-male fishes with the W chromosome produce a FAM-labeled 193-bp band and a biotin-labeled 147-bp band, of which the former will be captured by the control line, the latter will be captured by the test line (Fig. 2C). In summary, the results of RAA-CE and RAA-LFD for the 150 fishes analyzed here were completely consistent with those by anatomy and by classical PCR-based sex identification method, proving the effectiveness of our RAA-CE and RAA-LFD methods.

**Figure 4:**
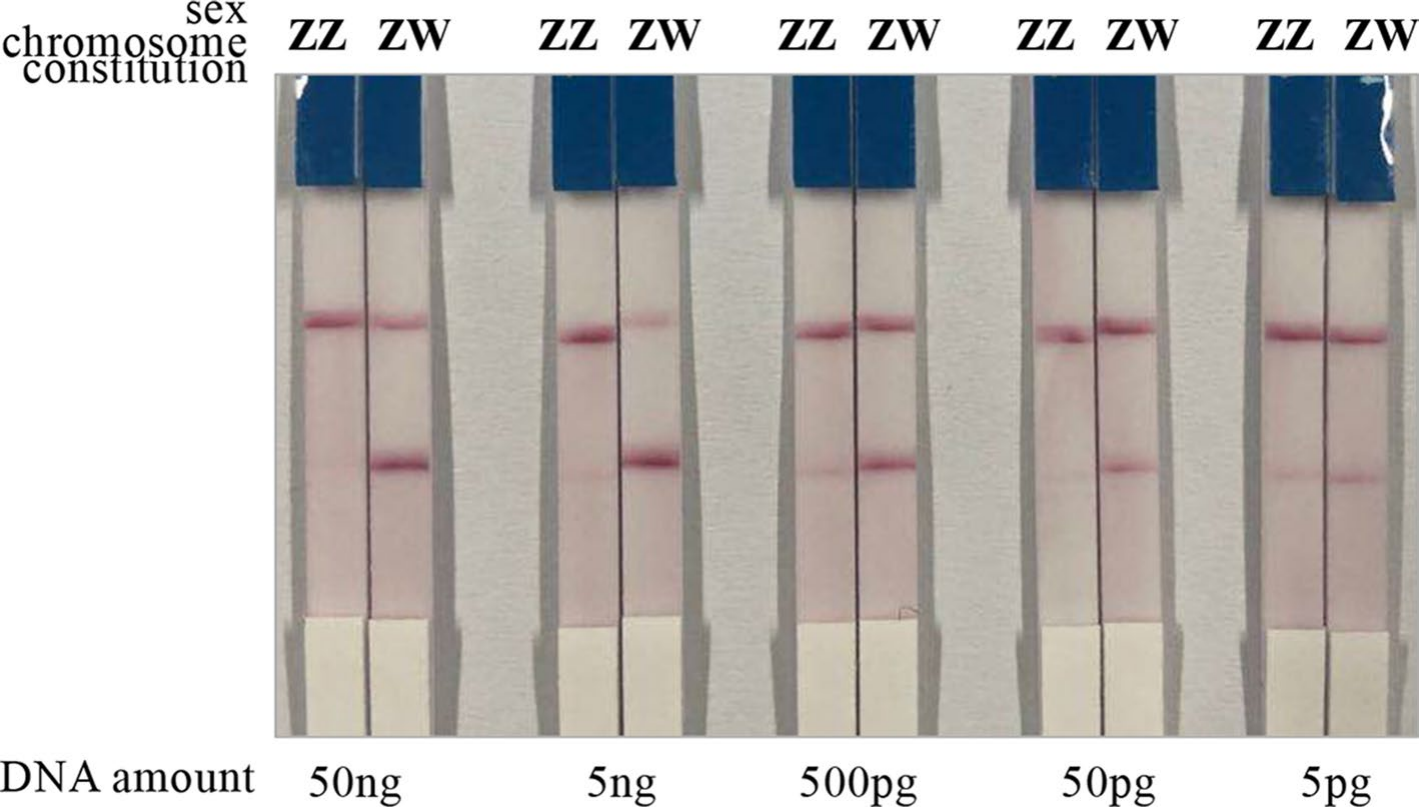
The data visualization of the RAA-LFD method using different amount of DNA as RAA templates. Five amounts of DNA, ranging from 5 pg to 50 ng, were tested for the RAA-LFD assay. Three replicates were performed for each of the DNA amount, with the representative LFD results shown here.

In addition, we performed the sex identification assay using series-diluted DNA samples of male and female fishes to further explore the limitation of our proposed RAA-LFD method. The 147-bp, W-specific RAA amplification product was clearly detected on LFD test strips when DNA amount per RAA reaction was at least 50 pg (Fig. 4). When the starting DNA amount was 5 pg per RAA reaction, the W-specific test band was too faint.

## Discussion

The RAA-CE and RAA-LFD methods are both effective for the sex identification of *C. semilaevis*. However, these two methods have different advantages. Capillary electrophoresis (CE) allows rapid (within 30 min) and high-throughput (96-sample per run) visualization, enabling distinguish between the short RAA amplicons. Moreover, CE is able to distinguish small differences in the size of amplification products, potentially eliminating limitations for primer/probe design. In contrast, taking the advantages of FAM-antibody and streptavidin–biotin interaction (Fig. 2C), LFD is simple and rapid than CE and other regular result visualization approaches, like gel electrophoresis. This feature of LFD helps to break through the boundaries of lab and offers opportunities for on-site tests.

We summarized and compared several technologies which fish sex identification methods could be based on, including PCR and electrophoresis, PCR and Sanger sequencing, RAA-CE and RAA-LFD methods as proposed in this study (Table 2). Most of the technologies require a modern molecular biology laboratory and many experimental instruments, and the operation for numerous samples will need well-trained, experienced technicians with a turn-around time of at least ~ 4 h (including reaction preparation, reaction time and detection of the results but the time for DNA extraction not included)^21, 35^. First, methods based on microsatellite markers or Sequence Characterized Amplified Region (SCAR) markers utilize PCR and electrophoresis technologies to identify sex and visualize the results, with an advantage of low cost but a disadvantage of time-consuming. Second, PCR and Sanger sequencing can be used for detecting sex-linked insertion/deletion and/or SNP polymorphisms in fishes and has the advantages of high accuracy, as sex-linked variations can be directly sequenced. However, this method also has several disadvantages, such as a long turnaround time and high costs for Sanger sequencing, either due to outsource of sequencing service or maintaining a sequencer in the laboratory. Last, RAA method can be coupled with CE or LFD visualization to detect fish sex. RAA has become one of the most widely used isothermal amplification technologies in microorganism pathogen detection due to several advantages: low-cost, timesaving, operationally simple, versatile while remaining the reaction specificity^33, 36–40^. Key features of RAA includes timesaving (20 ~ 40 min versus ~ 2 h for regular PCR; in our case, RAA takes 20 min) and cheaper costs for lab equipment (a dry bath for RAA versus thermal cycler for PCR), as RAA is performed at a constant temperature (usually 37 °C ~ 42 °C, 39 °C in our case). For result visualization, CE fits a lab-testing scenario as RAA amplification products usually are short and might not be distinguishable for electrophoresis-based detection, while LFD holds the potential that the RAA-LFD could be adapted to on-site applications, such as on-site testing in fishery fields. It is worth noting that the high-throughput and high accuracy of CE for batch survey also represents an advantage over the traditional gel electrophoresis method. In contrast to CE, LFD is timesaving and operationally simple: the only operation is to load RAA products onto the LFD test strip and read the control and test lines in 10 min. Besides, LFD also ensures specificity as it utilizes streptavidin–biotin capture to enrich specific amplification products (in our case, the ZW-specific RAA product). Overall, the RAA-LFD method proposed here combines the advantages of both technologies, provides a rapid and simple sex identification approach and is potential for on-site sex-identification, laying a foundation for molecular marker assisted (MAS) sex selection in aquaculture.

**Table 2:** Summary of different technologies for molecular identification of fish genetic sex.

Method	Time^a^	Estimated cost^b^	Required operational skills	Instruments required	Advantages	Disadvantages
PCR + electrophoresis	~ 4–6 h	$ 0.05 ~ 0.1	Regular molecular biology skills	Regular thermal cycler Gel imaging system	Low cost	Need to be performed in a regular molecular biology lab Time consuming
PCR + Sanger sequencing	~ 1 day	$ 3 ~ 8	Regular molecular biology skills Specialized skill for operating Sanger sequencer	Sanger sequencer, or Genetic Analyzer	Accuracy	High cost per sample Need to be performed in a molecular biology lab with an expensive Sanger sequencer^c^ Time consuming
RAA-CE	~ 30 min	$ 6.2^d^	Regular molecular biology skills Specialized skill for operating capillary electrophoresis	Dry bath with 96-well block	Time saving	High cost per sample Need to be performed in a molecular biology lab with an expensive CE
RAA-LFD	~ 30 min	$ 6.2^d^	Regular molecular biology skills	Dry bath with 96-well block	Time saving Convenience Potentially versatile testing scenarios	High cost per sample

^a^Estimated turnaround time of the sex identification methods. The time for reaction preparation, reaction and result visualization included, while the time for nucleotide extraction not included;

^b^Cost per reaction was estimated based on the prices of consumables and reagents in Anhui Province, China. Cost may vary depending on the sources of consumables and/or reagents;

^c^Outsourced Sanger sequencing usually lead to longer turnaround time (2–5 days);

^d^RAA-CE cost breakdown: the cost for RAA kit is $3.7 per sample; the cost for CE is $1.8 per sample; the cost for regular PCR reagent is $0.1 per sample; the cost for a set of labeled primer/probe is ~ $0.6 per sample. RAA-LFD cost breakdown: the cost for RAA kit is $3.7 per sample; the cost for LFD is $1.8 per sample; the cost for regular PCR reagent is $0.1 per sample; the cost for a set of labeled primer/probe is ~ $0.6 per sample.

LAMP, as another widely used isothermal amplification method^26^, has some different features when compared with RAA/RPA, while the lab equipment requirements and reaction time are generally comparable between the two methods. It has been suggested for sex or species identification for salmon and raptors^30, 31^. LAMP uses a DNA polymerase with a high activity of strand displacement, allowing an auto-cycling strand displacement DNA synthesis^26^. Unlike RAA that use a set of primers and a probe, LAMP uses a set of inner primers, a set of outer primers, and optional loop primers to enable the isothermal amplification. LAMP anneals at 60–65 °C and takes 30 to 90 min^29^. Key features of LAMP are that the method is highly sensitive and specific, with a wide adaptability to the downstream result visualization methods, including using gel electrophoresis, turbidity, pH-sensitive dyes, metal indicators and LFD. Based on these features, LAMP serves as an isothermal amplification method somewhat complementary to RAA. RAA is more expensive but more rapid and simple than LAMP, with high suitability for low-resource settings, while LAMP offers a highly sensitive, widely adaptable isothermal amplification with lower costs than RAA. In short, both methods open avenues to develop rapid isothermal amplification methods for fish sex identification, depending on species, targeted regions for primer design, costs, and application scenarios.

Besides the above-mentioned features of RAA-based methods, it is worth noting that RAA-based methods have some disadvantages. For example, RAA-based identification methods tend to have the false positive issue, which could be eliminated by careful selection of amplicon regions and stringent parameters for primers and/or probe. In our present study, the RAA-CE results for a few male fishes (lane 1, 2 and 3 in population 1, shown in Fig. 2B,C) showed faint bands around 100-bp, and these unspecific amplifications could be captured by LFD and faint test bands were also observed (Fig,2C). But possible unspecific amplification was not detected for most of the samples (Fig. 2 and Supplementary Fig. 1). For fish sex identification of *C. semilaevis*, better primers and probe without any non-specific amplification could be selected based restricted homology search using the *C. semilaevis* genome sequence^1^. Extending the RAA-LFD method to other fish species would require more experiments to evaluate and improve the specificity.

While the above-mentioned advantages of RAA-LFD and its effectiveness in fish sex identification as demonstrated herein, more experiments are needed to optimize the RAA-LFD method for different testing scenarios in future. First, DNA extraction methods compatible with suboptimal environments for on-site applications, such as fishery fields, needs to be developed. Since the focus of the present study was not to optimize all the experiment protocols for on-site testing, the DNA samples was extracted using the routine phenol–chloroform method in a modern lab. Recently, equipment-free DNA extraction has become feasible for plant and animal samples, as it is proved that Whatman No.1 filter paper can entrap and retain DNA due to its cellulose fibers^41^. DNA extraction from various animal samples, including human cell lines, blood, and pig lung swab, have been demonstrated successful by using such a method. More recently, this DNA extraction technique has been adapted to extract sea bass DNA infected with scale drop disease virus (SDDV) to develop isothermal amplification-based method for SDDV detection^42^, which provides a solid piece of evidence that simple, rapid, and on-site-compatible DNA extraction from fish samples is probable. Second, RAA amplification requires certain amount of DNA per reaction. Our results showed that at least 50 pg of DNA is required to amplify sufficient amount of the RAA product for LFD result visualization (Fig. 4). Third, the performance of RAA-LFD assay can be improved through optimization of the RAA primers, probe and reaction conditions. In our case, we were able to design the RAA probe with two SNPs between the Z and W genotypes, allowing distinguish between male and female fishes. In our opinion, it is plausible to identify other genomic regions more suitable for RAA primers and/or probe, which could contain more sequence variations between the Z and W genotypes and eliminate chances of non-specific amplification with higher specificity and robustness.

In this study, we successfully established RAA-based method for the sex identification of *C. semilaevis* based on a genomic region harboring multiple previously reported sex-linked markers. We combined RAA amplification with two distinct techniques for result visualization, CE and LFD, demonstrated the effectiveness of these methods. Particularly, RAA-LFD method features its advantages of operational simplicity, rapidity, and potential for on-site applications for fish sex identification.

## Materials and methods

### Animal samples

Two populations of *C. semilaevis* were used to test the performance of RAA-LFD sex identification method. One population is consisted of twenty-eight *C. semilaevis* fishes collected from the Bohai Sea by Freshwater Fisheries Research Institute of Fujian Province, China. The physiological sex of fishes was determined on the type of gonad as previously described^22^ and the physiological male and female fishes were divided. For population 1, the body weight and length of physiological males were 180.4 ± 1.2 g and 18.9 ± 1.0 cm, respectively, while the body weight and length of physiological females were 418.7 ± 0.8 g and 32.2 ± 0.9 cm, respectively. The population 2 of *C. semilaevis* consisted of 122 fishes were obtained from the Shengyi Fish Farming company (Tianjin, China). After separating physiological male and female fishes, the body weight and length of physiological males were 312.6 ± 6.9 g and 36.0 ± 2.0 cm, respectively, while the body weight and length of physiological females were 659.5 ± 7.9 g and 46.7 ± 2.4 cm, respectively. DNA was extracted from fins tissues (0.5 cm^2^) using the phenol–chloroform method^5^. The DNA samples were maintained in the lab at Microanaly, Inc. Anhui, China.

### Ethics statement

All fish handling and experimental procedures were approved by the Animal Ethical and Welfare Committee of Fujian Normal University. All experiments were carried out in accordance with the relevant guidelines and regulations. Study design and reporting followed the ARRIVE guidelines.

### Design of the recombinase-aided amplification (RAA) assay

Previously, the SSR marker (NCBI accession JN902162.1) for *C. semilaevis* sex identification was reported^2^. This marker targets the corresponding genomic regions at 8,935,873 bp ~ 8,936,194 bp and 18,076,313 bp ~ 18,076,651 bp for the W and Z sex chromosomes of *C. semilaevis*, respectively (NCBI accession numbers for the W and Z chromosomes are NC_024327.1 and NC_024328.1). An insertion/deletion polymorphism (IDP) marker (CS-SEX-3) and a SNP marker were further identified within this region and successfully applied for sex identification^22^.

To establish a RAA-based fish sex identification method, the intergenic region of ~ 1500 bp on the *C. semilaevis* sex chromosomes Z and W, where the SSR and IDP markers are located, wa chosen for RAA amplification in the present study. Within this region, a pair of RAA primers (F + R) were designed with the forward primer labeled by FAM at its 5’end (sequences shown in Table 1). An internal probe labeled by biotin at its 5’ end was designed to obtain a short amplicon (147 bp) specific on the W chromosome. To distinguish the W-specific 147-bp amplicon from other RAA amplfication products, 147-bp biotin-labeled amplicon can be captured by the streptavidins on the test line of LFD. By contrast, the 228-bp and 193-bp amplicons from the Z and W chromosome (Fig. 2B,C), respectively, are FAM-labeled due to the primer F and can be bound by anti-FAM antibody-colloidal gold when they flow through the sample application area of the LFD. Then these RAA products can be captured by the anti-rabbit antibody on the control line of LFD and the control line are visible (Fig. 2C). To facilitate RAA result visualization, we proposed two methods with different advantages: one is RAA-capillary electrophoresis (RAA-CE), and the other is RAA-lateral flow dipstick (RAA-LFD).

The design of the RAA primers and probe and the selection of the amplicon region follow these requirements: (1) the amplicon should be between 100 to 500 bp, with a preferred length between 100 to 250 bp; (2) the amplicon region should avoid direct/inverted repeats and palindromes with a preferred GC content between 40 to 60%; (3) The length of primers should be between 30 and 35 bp with a GC content between 30 and 70%; shorter primers could decrease the reaction speed and/or sensitivity; (4) When design the primer, long tracks of G should be avoided at the 5′ end, and G/C are recommended at the 3′ end; (5) The melting temperature, secondary structure, dimer formation should be evaluated by a primer design software. Secondary structures and primer dimers should be minimized, and mismatch between the primer and template should be avoided; (6) For the probe design, specificity should be the top consideration and the probe and primers should not be overlapped; (7) the probe should not form dimer with the primers; (8) The specificity of both primer and probe should be evaluated using BLAST against the target genome (in this case, the *C. semilaevis* genome).

Following the above-mentioned criteria, the 30-bp forward and reverse primers (namely F and R) were designed within the sex-linked genomic region for the RAA saay using Primer3Plus (http://primer3plus.com)^43^. The internal probe was designed taking advantage of the two SNPs differing the W-chromosome allele from the Z-chromosome allele to specifically amplify a 147-bp product from the W chromosome (the SNPs labeled by asterisks in Fig. 2A). Primer F is FAM labeled at its 5’ end, while the internal probe is biotin labeled at its 5’ end. The primers and probe are provided (Table 1). The primers and probe were synthesized from TsingKe, Inc. (Nanjing, China). The design of the primers and internal probe has two advantages: (1) the amplicon region was purposefully selected to have several InDels so that the RAA products amplified by primer F and R differ in size between the Z and W chromosomes (228-bp versus 193 bp); (2) a W-chromosome specific amplicon (147 bp) can be obtained by primer F and the internal probe, facilitating distinguishment between fish genetic sex; (3) the biotin label allows the 147-bp W-specific amplicon to be captured by the test line of LFD, while the other RAA products specifically amplified from the sex marker region can be captured by the control line of LFD due to the FAM label.

### RAA assay

The RAA reaction was performed using the RAA Nucleic Acid Amplification kit following the manufacturer’s instructions (product no. B00000, from Jiangsu Qitian Gene Biotechnology Co., Ltd, Jiangsu, China; information on the kit available at: http://en.qt-bio.com/?opt=product&optId=78&nid=484). RAA has an amplification mechanism similar to RPA, which requires three key proteins to displace double-stranded DNA template and to hybrid primers with template: a recombinase, a recombinase loading factor, and a single-stranded binding protein (SSB). Unlike RPA, RAA employs recombinase UvsX (from *E.coli*) and SSB but doesn’t require the recombinase loading factor (Supplementary Fig. 1)^34^. Recombinase forms a nucleoprotein complex with a primer/probe. The complex searches for homologous sequences in double-stranded DNA (template) and hybridizes with the DNA. The primer hybridizes with the template strand using a strand exchange mechanism, while the complementary strand become single and stabilized by the SSB protein. After primer hybridization, the recombinase complex disassembles and form another nucleoprotein with a new primer molecule, cycling the process of template recognition and hybridization. Once the primer is incorporated in the double-stranded DNA, DNA polymerase initiates the synthesis at the 3’ end of the primer. DNA synthesis starts from both directions simultaneously when both forward and reverse primers incorporated with the template. Exponential amplification then starts as a regular PCR but occurs in the isothermal condition.

The volumes of 10 μM primers and 10 μM internal probes were adjusted following the manufacture’s recommendations. The 50-μL RAA reaction contains 1 × rehydration buffer (25 μL), 50-ng DNA template, 2 μL of each primer, 0.5 μL of the probe, and 2.5 μL of 280-nM magnesium acetate. The magnesium acetate was pipetted into tubes to initiate reaction. The reaction was transferred to a dry bath with a 96-well block and incubated at 39 °C for 20 min. After the incubation, 50 μL of phenol/chloroform was added to terminate the reaction. The reaction was then centrifuged for 30 s in a mini-centrifuge. The supernatant was transferred to another tube for result visualization.

### Visualization of RAA products using CE and LFD

Both capillary electrophoresis (CE) and lateral flow dipstick methods were used to detect the RAA products. For the CE visualization, the RAA products were purified by using VAHTS DNA clean beads (Vazyme, China). The quantity and quality of the RAA products were detected by using capillary electrophoresis (Agilent 4200, State of California, USA).

The RAA products was also detected using LFD assay (LFD test strip #JY0301 from Tiosbio, Beijing, China). Briefly, 5-μL of the RAA amplicons and 95-μL of the diluent buffer were mixed adequately in a 1.5-mL centrifuge tube. The LFD sample application area was then directly dipped into the RAA product-buffer mixture. Due to capillary force, the RAA product-buffer mixture flew through the LFD sample application area where the anti-FAM antibody-colloidal gold was bound with the RAA amplicon (including the 147-bp, 193-bp and 228-bp products). Due to the streptavidin–biotin interaction, only the 147-bp W-specific amplicon was captured on the test line of LFD and the test line emerged. The other two amplicons that had been bound by the immuno gold were then captured by the anti-rabbit antibody on the LFD control line and the control line emerged (Fig. 2). The LFD results were ready for observation within 10 min.

To determine the minimum amount of DNA template that is required for efficient RAA amplification, DNA samples were series diluted from 50 ng per reaction, to 5 ng, 500 pg, 50 pg, and 5 pg per reaction. The series diluted DNA samples were subjected to the RAA-LFD assay. Three random DNA samples were used as replicates.

### Validation of RAA-based sex identification by using the IDP marker

A previously reported PCR-based genotyping method was used for validation of our RAA method^22^. The PCR-based genotyping method uses the insertion/deletion polymorphism marker (CS-SEX-3F: GCAGCAACCACATCCTCAGT and CS-SEX-3R: CAGGAACATGCAGTAGGACA). The PCR amplification was performed in a 25-μL reaction containing 50 ng of genomic DNA using the program as follows: 94 °C for 5 min, 35 cycles of 94 °C for 30 s, 60 °C for 30 s, and 72 °C for 1 min, with a final extension for 7 min. The original image of the electrophoresis gel for Fig. 2A is provided as Supplementary Fig. 2.

## Supplementary Information

Supplementary Figures.
