# Supplementary material for: A simple and rapid method for fish sex identification based on recombinase-aided amplification and its use in Cynoglossus semilaevis
Source: Sci Rep. 2021 May 17;11:10429. doi: 10.1038/s41598-021-89571-z (PMC8128863; doi:10.1038/s41598-021-89571-z)
Supplement: Supplementary file 1 — Supplementary Figures. [file 41598_2021_89571_MOESM1_ESM.pdf]

A simple and rapid method for fish sex identification based on recombinase-aided amplification and its use in *Cynoglossus semilaevis*

Zhichao Nie [#2,3], Peng Lü [#2,3], Rusong Zhang [2,3], Yishuai Tu [3], Zhenni Liu [3], Yin Li [4,7], Cong Tang [3], Xiqing Li [3], Kun Zhao [3], Qiang Zhou [3], Feng Li [3], Jun Wang [3], Zhanzhuang Zeng [5,6,*], Min Tu [4,*] and Hong Zhang [1,3,*]

[1] Anhui Provincial Key Laboratory of the Conservation and Exploitation of Biological Resources, College of Life Sciences, Anhui Normal University, Wuhu 241000, China;
[2] Institute of Life Sciences, Jiangsu University, Zhenjiang 212013, China;
[3] Anhui Microanaly Gene Co., Ltd., Hefei 230601, China;
[4] Waksman Institute of Microbiology, Rutgers, The State University of New Jersey, Piscataway, NJ 08854, USA;
[5] Freshwater Fisheries Research Institute of Fujian Province, Fuzhou 350002, China;
[6] College of Life Sciences, Fujian Normal University, Fuzhou 350002, China;
[7] Present address: The Genetic Engineering International Cooperation Base of Chinese Ministry of Science and Technology, the Key Laboratory of Molecular Biophysics of Chinese Ministry of Education, College of Life Science and Technology, Huazhong University of Science & Technology, Wuhan 430074, China.

# These authors contributed equally to this work.
***** Correspondence:
zhanghong@magiltd.com; Tel.: +86-0551-82311173 (H.Z.);
tumin.ly@hotmail.com; Tel.: +01-848-445-6446 (M.T.)

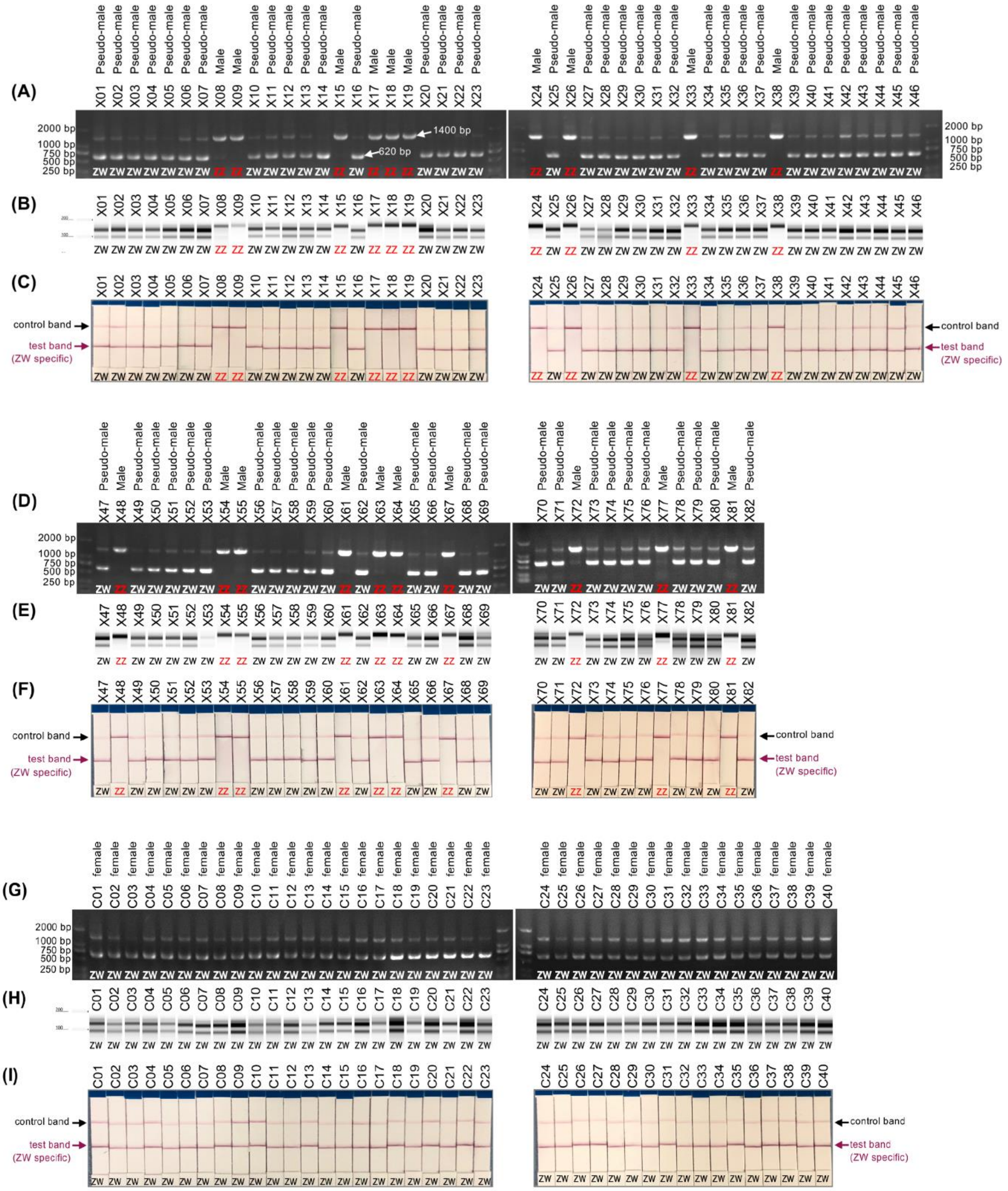

Supplementary Figure 1. The sex identification results for population2.

First, the genetic sex of each *C. semilaevis* individual from population 2 was determined by PCR-based method with the IDP marker from a previous report[22] (Fig.S2A, S2D, S2G). Second, the genetic sex of each *C. semilaevis* individual from population 2 was determined using RAA-CE (Fig.S2B, S2E, S2H). CE results showed a single band of 228-bp for the male fishes (ZZ karyotype) and three bands for the female and pseudo-male fishes, including a 193-bp band and a 147-bp band specific for the ZW karyotype. Third, the genetic sex of each *C. semilaevis* individual from population 2 was determined using RAA-LFD (Fig.S2C, S2F, S2I). LFD visualization effectively captured and separated the W-specific, 147-bp RAA products from those bands amplified by the primer F and R (228-bp and 193-bp).

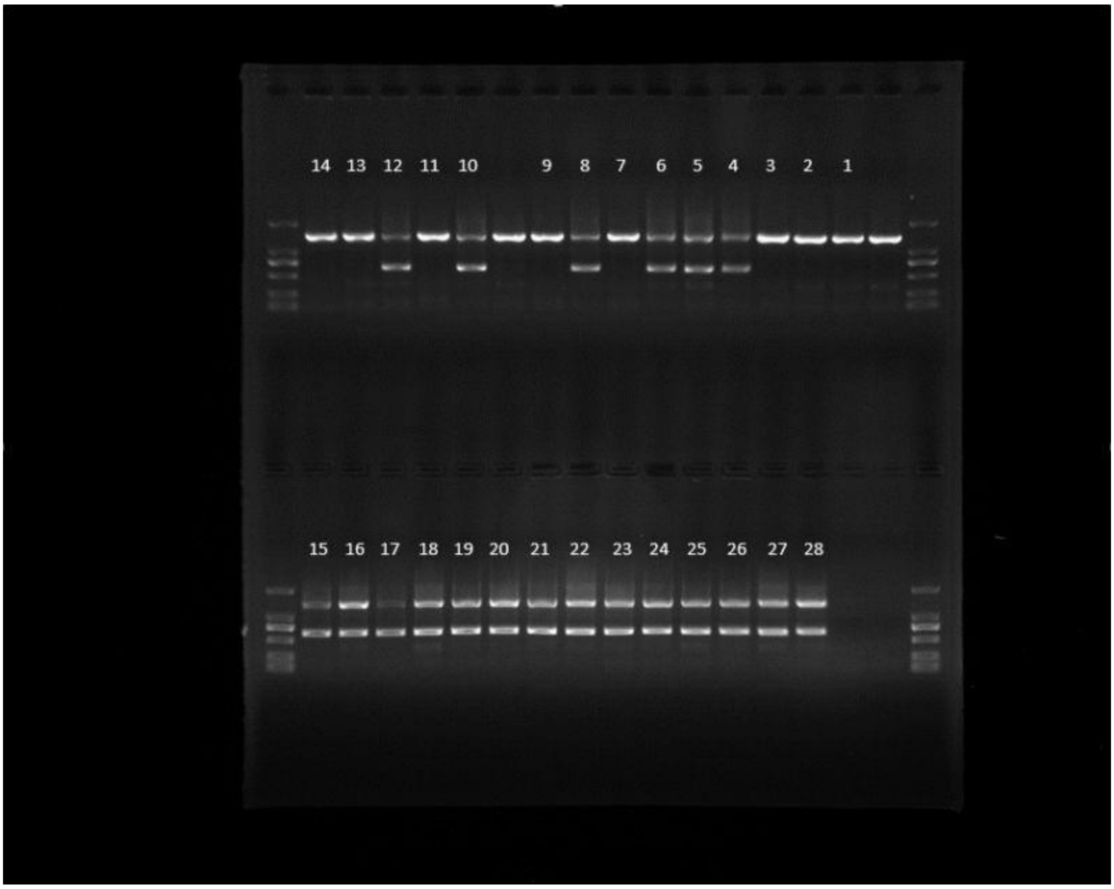

Supplementary Figure 2. The original image of electrophoresis gel for Figure 3A. The lanes are labeled in accordance with Figure 3A.
